# Supplementary material for: Empirical methods for controlling false positives and estimating confidence in ChIP-Seq peaks
Source: BMC Bioinformatics. 2008 Dec 5;9:523. doi: 10.1186/1471-2105-9-523 (PMC2628906; doi:10.1186/1471-2105-9-523)
Supplement: Additional File 1 — A variety of html documents from the USeq web site detailing the available applications, their best usage, output file type descriptions, command line menus, etc. [file 1471-2105-9-523-S1.zip › USeqUserGuides/JavaDoc.zip!/JavaDoc/util/gen/package-summary.html]

util.gen


|  |  |  |
| --- | --- | --- |
| **Overview** \| **Package** \| Class \| **Use** \| **Tree** \| **Deprecated** \| **Index** \| **Help** | |  |
| **PREV PACKAGE**   **NEXT PACKAGE** | **FRAMES**    **NO FRAMES**     **All Classes** |  |


---

## Package util.gen

Variety of helper methods for Email, IO, Encryption, HTML, Swing...

**See:**
  
          **Description**

| **Class Summary** | |
| --- | --- |
| **Bin** | Info and methods about a particular bin, used by the Histogram class. |
| **CubicSpline** | For calculating cubic splines of incremental data, interpolation, from http://www.frank-buss.de/CubicSpline.java |
| **Email** |  |
| **FileSize** |  |
| **Histogram** | Generates histograms. |
| **HTML** | Static methods for HTML related tasks. |
| **IO** | Static methods for Input Output related tasks. |
| **Job** |  |
| **LinearRegression** | For calculating a simple linear regression. |
| **MD5** | This class implements the MD5 algorithm as described in RFC1321. |
| **MD5Crypt** | This class defines a method, `crypt()`, which takes a password and a salt string and generates an OpenBSD/FreeBSD/Linux-compatible md5-encoded password entry. |
| **Misc** | A variety of static methods. |
| **Num** | Math and statistical static methods. |
| **Passwords** | Methods for generating one time passwords. |
| **PearsonCorrelation** | Calculates Pearson Correlations (R) in two ways, using a straight static method on two arrays or by 1st adding paired arrays sequentially and 2nd calculating the correlation on the cumulative sum. |
| **RankedFloatArray** |  |
| **RankSample** |  |
| **RankSampleIndexComparator** | Sorts by index, small to large. |
| **RankSampleValueComparator** | Sorts by value, small to large. |
| **ReadZip** |  |
| **SpearmanCorrelation** | Calculates a Spearman correlation coefficient (rho), in the case of ties, averages the ranks and returns a Pearson correlation coefficient on the ranks. |
| **SQL** | Utility class to hold database and relevant fields and methods, some from Hall and Brown, most from BioRoot. |
| **Swing** | Methods related to Swing apps. |
| **TextFrame** | Creates a free standing plain editable text window. |
| **XML** | Utilities for XML parsing. |

## Package util.gen Description

Variety of helper methods for Email, IO, Encryption, HTML, Swing...

---


|  |  |  |
| --- | --- | --- |
| **Overview** \| **Package** \| Class \| **Use** \| **Tree** \| **Deprecated** \| **Index** \| **Help** | |  |
| **PREV PACKAGE**   **NEXT PACKAGE** | **FRAMES**    **NO FRAMES**     **All Classes** |  |


---
